# International Congress on Transposable Elements (ICTE) 2012 in Saint Malo and the sea of TE stories

**DOI:** 10.1186/1759-8753-3-17

**Published:** 2012-10-30

**Authors:** Abdelkader Ainouche, Mireille Bétermier, Mick Chandler, Richard Cordaux, Gaël Cristofari, Jean-Marc Deragon, Pascale Lesage, Olivier Panaud, Hadi Quesneville, Chantal Vaury, Cristina Vieira, Clémentine Vitte

**Affiliations:** 1 UMR-CNRS 6553 Ecobio, Université de Rennes-1, Campus de Beaulieu Bât 14A, Rennes, F-35042, France; 2 CNRS, Centre de Génétique Moléculaire, UPR3404, 1 avenue de la Terrasse, Gif-sur-Yvette, F-91190, France; 3 Département de Biologie, Université Paris-Sud, CNRS UMR 8621, 15 rue Georges Clémenceau, Orsay, F-91405, France; 4 Laboratoire de Microbiologie et Génétique Moléculaires, Centre National de Recherche Scientifique, Unité Mixte de Recherche 5100, 118 Rte de Narbonne, Toulouse, F-31062, France; 5 Université de Poitiers, CNRS UMR 7267 Ecologie et Biologie des Interactions, Equipe Ecologie Evolution Symbiose, 40 avenue du Recteur Pineau, Poitiers, F-86022, France; 6 Institute for Research on Cancer and Aging in Nice (IRCAN), CNRS UMR 7284, INSERM U1081, University of Nice-Sophia Antipolis, Faculty of Medicine, 28 avenue de Valombrose, Nice, F-06107, France; 7 UMR CNRS 5096 Laboratoire Génome et Développement des Plantes, Université de Perpigan, CNRS LGDP 58 avenue Paul Alduy, Perpignan, F-66860, France; 8 CNRS Université Paris Diderot UMR7212, INSERM U944, Laboratoire Pathologie et Virologie Moléculaire, Institut Universitaire d’Hématologie, Hôpital St Louis, 1 avenue Claude Vellefaux, Paris, F-75010, France; 9 INRA, UR 1164, URGI, Unité de Recherche en Génomique-Info, Rte de Saint-Cyr, Versailles, F-78026, France; 10 UMR/CNRS 6293, Clermont Université, INSERM U1103, Faculté de Médecine, 28 Place Henri Dunant, Clermont-Ferrand, F-63000, France; 11 Laboratoire de Biométrie et Biologie Evolutive, CNRS UMR5558, Université Lyon 1, Université de Lyon, 43 Bd du 11 novembre 1918, Villeurbanne, F-69622, France; 12 CNRS, UMR de génétique végétale, Ferme du Moulon, Gif-sur-Yvette, F-91190, France

**Keywords:** Transposable elements, Evolution of transposable elements, Impact on genomes, Control of transposition, Mechanisms of transposition

## Abstract

An international conference on Transposable Elements (TEs) was held 21–24 April 2012 in Saint Malo, France. Organized by the French Transposition Community (GDR Elements Génétiques Mobiles et Génomes, CNRS) and the French Society of Genetics (SFG), the conference’s goal was to bring together researchers from around the world who study transposition in diverse organisms using multiple experimental approaches. The meeting drew more than 217 attendees and most contributed through poster presentations (117), invited talks and short talks selected from poster abstracts (48 in total). The talks were organized into four scientific sessions, focused on: impact of TEs on genomes, control of transposition, evolution of TEs and mechanisms of transposition. Here, we present highlights from the talks given during the platform sessions. The conference was sponsored by Alliance pour les sciences de la vie et de la santé (Aviesan), Centre national de la recherche scientifique (CNRS), Institut national de la santé et de la recherche médicale (INSERM), Institut de recherche pour le développement (IRD), Institut national de la recherche agronomique (INRA), Université de Perpignan, Université de Rennes 1, Région Bretagne and Mobile DNA.

**Chair of the organization committee:**

Jean-Marc Deragon

**Organizers:**

Abdelkader Ainouche, Mireille Bétermier, Mick Chandler, Richard Cordaux, Gaël Cristofari, Jean-Marc Deragon, Pascale Lesage, Didier Mazel, Olivier Panaud, Hadi Quesneville, Chantal Vaury, Cristina Vieira and Clémentine Vitte

## Introduction and keynote lecture

### Jean-Marc Deragon

Transposable elements (TEs) are truly fascinating biological entities. This is, of course, the opinion of all ICTE 2012 participants who met last April in Saint Malo, France, for what turned out to be a very exciting congress. A major evolution in the field since the ICTE 2008 edition is the routine use by many research groups of next-generation sequencing methods. This technological revolution has not only allowed significant progress in our fundamental understanding of TE biology, but has also revealed more than ever the importance of TE research to better understand genome evolution and to decipher the complexity of gene expression networks. The keynote lecture of the meeting, given by **Jef Boeke** (John Hopkins University, Baltimore, MD, USA), set the tone and gave a very good example of what studying TEs can lead to these days. First, he explained how massive sequencing could be used to understand in great detail basic aspects of retrotransposon behavior. Using deep sequencing of yeast Ty1-flanking sequence amplicons, he was able to reveal not only that the majority of Ty1 insertions were associated with the 5′ regions of RNA polymerase III transcribed genes, but also that an exquisitely specific relation exists between Ty1 insertion sites and nucleosomal DNA segments at specific positions on the nucleosome lateral surface. The use of massive sequencing technologies was essential to observe this specific relation and led to the conclusion that a dynamic process such as nucleosome remodeling is likely involved in Ty1 integration. Next, he illustrated the power of deep sequencing to carry out screens of human genomes for retrotransposon insertion polymorphisms (RIPs) and found that frequencies of individual presence/absence alleles vary dramatically and could be used to characterize specific human populations. Finally, he argued that understanding TEs is essential to generate a completely synthetic yeast genome lacking all destabilizing elements. In the long term, yeasts containing such a synthetic genome could be used to evaluate the impact on genome evolution and fitness of reintroducing different types of TEs. Genome-wide approaches have been enthusiastically adopted by many participants and adapted to study the impact of TEs on genome and gene expression to better understand the control of TEs, their evolution and their mechanism of transposition.

## Session 1: Impact on genomes

### Olivier Panaud, Hadi Quesneville, Clémentine Vitte

Genome-wide TEs analysis in plants and animals shows that TEs are a major and extremely active component of eukaryotic genomes, constituting up to 90% of the nuclear DNA in some species (for example bread wheat). Their insertion and deletion activities are affected by factors such as chromatin structure, recombination rate, and gene density. **Henry Levin** (National Institutes of Health, Bethesda, MD, USA) described a novel approach to identify the function of eukaryotic genes based upon dense maps of transposon integration. As a proof of concept, he generated a large library of transposon Hermes insertions in *Schizosaccharomyces pombe*. Cells with insertions in genes important for growth become depleted in the culture. Deep sequencing of insertion sites reveals which insertions cannot be tolerated under a specific growth condition, determining which genes are required for cells to grow in defined medium.

TEs contribute to genomic variation between individuals in populations. In human, while most retroelements are remnants of ancient transposition events and are no longer capable of active retrotransposition, a fraction remains active and contributes to variation between individuals. These elements belong almost exclusively to the Alu, LINE-1 (L1), and SVA families of non-long terminal repeat (LTR) retrotransposons. The 1000 genome project, the goal of which is to provide a comprehensive resource on human genetic variation through sequencing genomes from different populations, offered **Mark Batzer** (Louisiana State University, Baton Rouge, LA, USA) the opportunity to analyze polymorphisms related to these three active elements. This revealed 7,380 polymorphic mobile element insertions. Experimental validation showed that while the small Alu1 element (300 bp) has a low false detection rate (5%), a higher error rate is found for the several kilobase long L1 and SVA elements (31% and 25%, respectively), a feature that may be due to the use of short reads and low genome coverage. Characterization of mobile subfamilies through analysis of target site duplication length distributions revealed that the AluYa5 subfamily accounts for the vast majority of Alu variation and is therefore the main driver of human Alu insertion.

The growth of genomic data made available by next-generation sequencing (NGS) technologies has opened new perspectives for studying TE evolutionary dynamics across many lineages through comparative studies. This necessitates robust methods for TE identification and most importantly their classification from sequenced genomes. **Moaine Elbaidouri** (Laboratoire Génome et Développement des Plantes, Perpignan, France) developed a new bioinformatic procedure for mining and clustering of LTR retrotransposons that allows comprehensive comparative genome-wide study of the impact of retrotransposition on genome structure in the plant kingdom. Regardless of the size of the plant genome, only a few families have undergone recent transposition. Moreover, difference in genome size is correlated with the extent of the burst of the most recent active families. This also suggests that most ancient TE insertions have been eliminated, confirming that differentiation of the non-genic compartment of plant genomes occurs through a very fast turnover of TE-related sequences.

**Philippe Glaser** (Institut Pasteur, Paris, France) introduced an original transposition mechanism observed in *Streptococcus* insertion sequence (IS) TEs. After excision, the TE circles are infectious as they can be transmitted from one cell to another during conjugation. Interestingly, the integration is specifically targeted into the promoter region of genes, limiting the impact of this TE on the genome.

**Aurélien Doucet** (laboratory of John Moran, University of Michigan Medical School, Ann Arbor, MI, USA) discussed about the identification of an unconventional mechanism for the human LINE-1 ORF2 translation. Using biochemical and cellular approaches as well as the L1 retrotransposition assay, developed in the laboratory of John Moran, he described the translation of ORF2, located downstream of ORF1 in the L1 bicistronic transcript, likely occurring by termination/re-initiation. This mechanism seems specific to the bicistronic context. Deletion of the inter-ORF spacer or substitution of the first ORF still lead to ORF2p expression; indicating that there is likely no specific sequence upstream of ORF2 to allow its translation.

TEs are also responsible for a large proportion of visible mutations and possibly a substantial proportion of advantageous mutations. Many TEs are located within or near genes and have been shown to play important roles in gene regulation and function. **Helen Rowe** (laboratory of Didier Trono, Ecole Polytechnique Fédérale de Lausanne, Lausanne, Switzerland) showed that endogenous retroviruses (ERVs) are transcriptional landmines whose KAP1-mediated control is essential to preserve transcriptional networks at the heart of embryonic stem (ES) cell pluripotency. Regulation of retroelements is, therefore, critical not just to prevent retrotransposition, but more broadly to safeguard the timely activation of genes through development.

**Matthias Zytnicki** (laboratory of Hadi Quesneville, INRA, Versailles, France) noted that for some TE families, small regions that seem to be over-represented in the genome might contain putative transcription start sites (TSSs). This could imply that some specific TE fragments may have been positively selected by the host genome. He showed that active transposable copies influence host gene transcription and that TSSs of TEs have been exaptated to provide new gene structures.

As advantageous mutations, TEs have been involved in the domestication of several crop species. **Eugenio Butelli** (Norwich Research Park, Norwich, UK) gave a spectacular example of the impact of LTR retrotransposons in the occurrence of blood oranges. Following characterization of the *ruby* gene - which encodes the conserved domain of the Myb regulators of anthocyanin biosynthesis - from the Sicilian Moro blood orange, the authors found that this gene is expressed in the fruit of several blood oranges but not in that of the common blond oranges. The difference in expression was shown to be driven by insertion of a copia-like LTR retrotransposon in the 5′ untranslated region **(**UTR**)** of the gene of the blood orange. The 3′ LTR of the retrotransposon contributes a transcription initiation site that is not present in blond oranges. This creates a chimeric LTR/gene transcript, which leads to gene expression. Interestingly, both complete element and solo LTR forms of the element, which are found in different Sicilian blood orange varieties, can induce gene expression. Finally, analysis of *ruby* in an ancient Chinese blood orange variety of non-Sicilian origin revealed the insertion of a different LTR retrotransposon in its 5′ UTR. This insertion is in inverse orientation and the retrotransposon does not bring a transcription start site through its LTR. The observation of two independent LTR retrotransposon insertions inducing the expression of the *ruby* gene through different mechanisms is particularly striking.

TEs are also clearly involved in building host gene regulation. Through a chromatin immunoprecipitation followed by massive DNA sequencing **(**ChIP-seq) approach, **Guillaume Bourque** (McGill University and Genome Quebec Innovation Center, Montreal, QC, Canada) showed that, in mammals, some repeats have a strong association with transcription factors. To investigate the presence of TE-based transcriptional regulatory networks, the authors analyzed whether TE-embedded sites are associated with genes regulated by the protein, which are binding these sites. Through a simultaneous analysis of protein occupancy and expression data, they revealed that different TE families have contributed to a significant fraction of binding sites in both human and mouse. Although orthologous genes had the same regulation pattern in both human and mouse, the type of involved binding sites were different, revealing that TEs have contributed in rewiring gene expression network in mammals.

Beyond being potential mutagenic agents, TEs are sometimes co-opted for functional roles in eukaryotic genomes. Recent results indicate their central role in epigenetic control of gene expression. In the mouse, retrotransposons are often located in repressed regions and are thought to induce heterochromatin formation and its spread. However, direct evidence for TE-induced local heterochromatin in mammals is surprisingly scarce. To examine this phenomenon, **Rita Rebollo** (laboratory of Dixie Mager, British Columbia Cancer Agency, Vancouver, BC, Canada) analyzed insertionally polymorphic sites for three retrotransposons (IAP, ETn/MusD, and LINE) at specific loci in two mouse embryonic stem cell lines and investigated the chromatin state around the full and empty sites using ChIP-seq analysis. While IAP elements induce H3K9me3 heterochromatic marks in flanking genomic DNA, such heterochromatin is not induced by LINEs and only by a minority of polymorphic ETn/MusD copies. Only one case of transcriptional silencing via IAP-induced heterochromatin was identified. DNA methylation analysis showed no difference between full and empty sites for IAP, but revealed that MusD induces DNA methylation at short distances (within 1.5 kb). On the other hand, when the two LTRs of a given element are compared, the LTR located the closest to the gene is not methylated, whereas the other is, suggesting that genes can also influence MusD methylation. While influence can be seen in both directions (retrotransposon to gene or gene to retrotransposon), many cases of equilibrium exist. The role of insulator protein is proposed as a wall to prevent spreading between genes and retrotransposons.

**Prescott Deininger** (Tulane University, New Orleans, LA, USA) created a novel cassette-based recombination assay system allowing evaluation of a broad range of potential Alu-related sequence factors. Replacing one of the Alu elements with sequences having different percentage and distribution of mismatches demonstrates a strong dependence on sequence identity, as well as distribution. With perfectly matched Alu elements, the system detects only Alu/Alu non-allelic homologous recombination (NAHR) events. However, as mismatch is increased, deletions caused by non-homologous end joining (NHEJ) become dominant. Surprisingly, at moderate levels of mismatch, both NAHR and NHEJ recombination are suppressed, suggesting a complicated local interference between DNA repair pathways.

## Session 2: Control of transposition

### Pascale Lesage and Chantal Vaury

TEs are tightly regulated to prevent potential damage they might cause to the host genome if their activity is too high. A variety of regulatory systems has been selected by the cell to counteract the mutagenic effects of TEs. A given TE uses a set of mechanisms to control its activity at several levels. These may vary in detail, even between TEs that are phylogenetically related or between different tissues within an organism. Control of transposition activity can involve autoregulatory circuits or interactions with cellular factors. It can occur at different stages of transposition such as transcription, translation, transport, intracellular formation of nucleoprotein structures and efficiency/selectivity of their integration into the cell genome. TE activity can also be silenced by different epigenetic regulation mechanisms: at the transcriptional level via DNA methylation and/or histone modifications, and/or at post-transcriptional level via small RNAs. The talks in the ‘Control of transposition’ session revealed the ever-expanding diversity of mechanisms that control transposition activity. Each talk focused on different TE models (ranging from LTR and non-LTR retrotransposons to DNA transposons) and in several organisms (yeast, Drosophila, mouse, human).

The transcription of the Ty1 LTR retrotransposon of *Saccharomyces cerevisiae* is activated by environmental stresses through host-cell signaling pathways and produces Ty1 sense and antisense RNAs. **Pascale Lesage** (CNRS, INSERM, Institut Universitaire d’Hématologie, Paris, France) reported that Ty1 transcription increases in response to a severe deficiency in intracellular ATP and ADP levels. The Tye7 transcription factor originally identified as an activator of Ty1-adjacent gene transcription, is induced in response to ATP/ADP depletion, inhibits Ty1 antisense transcription and activates Ty1 mRNA transcription. Tye7 probably represses Ty1 antisense RNA synthesis, directly. These findings highlight a new level of control of Ty1 mobility by environmental stress conditions, since Ty1 antisense RNAs play an important role in regulating Ty1 at both the transcriptional and post-transcriptional stages.

LTR retrotransposon RNA within virus-like particles (VLPs) serves as the genome, as the template for translation of Gag and Pol proteins, and for reverse transcription. An important issue concerns the partition of RNA among these three functions. **Joan Curcio** (Wadsworth Center, Albany, NY, USA) described the use of selective 2'-hydroxyl acylation analyzed by primer extension (SHAPE) analysis to resolve the secondary structure of *in vitro*-transcribed Ty1 RNA (nucleotide 1–380) that is necessary for packaging and initiation of translation and reverse transcription. This structure predicts the existence of a pseudoknot separating the RNA into two well-organized domains. Mutational analysis suggests that the pseudoknot is a determinant of Ty1 RNA packaging and retrotransposition, which may delineate functional as well as structural domains in Ty1 RNA. J. Curcio also characterized several cellular factors required for the formation of retrosomes, microscopically distinct foci where retrotransposon-encoded RNA and proteins localize, inside the yeast cell. Although these cofactors are not necessary for Ty1 RNA and Gag protein expression or VLP formation, Ty1 cDNA is strongly reduced in their absence, suggesting that retrosome formation may be a required step in the production of replication-competent VLPs.

The L1 non-LTR retrotransposon is the most abundant and active mobile element in the human genome. About 34% of the human genome is a consequence of L1 element activity. Moreover, L1 has been shown to cause genetic disorders and tumorigenic diseases by local genomic destabilization. Two talks focused on how mammalian cells have adopted several strategies to restrict L1 mobility and potentially deleterious consequences of its uncontrolled retrotransposition. L1 replication by target-primed reverse transcription of its RNA generates a 3' flap intermediate at the insertion site, resulting from the elongation of the L1 cDNA. **Geraldine Servant** (laboratory of Prescott Deininger, Tulane University, New Orleans, LA, USA) showed that the DNA-repair endonuclease complex ERCC1-XPF, which specifically recognizes and cleaves 3′ flap structures, and core proteins of the nucleotide excision repair (NER) pathway, such as the XPD helicase and the lesion-binding protein, XPC, limit L1 retrotransposition. One hypothesis is that the NER pathway recognizes an L1 insertion intermediate as damage, disturbing the helical structure of the DNA, and recruits ERCC1-XPF to inhibit subsequent insertion. These data point to a new function for NER in the maintenance of the human genome integrity by controlling L1 proliferation.

Human APOBEC3 (A3-A to A3-G) cytidine deaminase proteins also contribute to intracellular defense against retroelements and inhibit L1 retrotransposition. However, genomic L1 retrotransposition events that occurred in the presence of L1-inhibiting A3 proteins are devoid of expected G-to-A hypermutations in L1 DNA, suggesting a deaminase-independent mechanism that restricts L1. **Gerald Schumann** (Paul-Ehrlich-Institut, Langen, Germany) showed that A3C-mediated L1 restriction requires both an intact dimerization site and a functional RNA-binding pocket domain of the protein. He found that L1 ORF1p and A3C are located in the same fraction after density gradient centrifugation of L1 ribonucleoprotein particles (RNPs). Co-immunoprecipitation experiments confirmed a direct interaction of L1 ORF1p and A3C. L1 element amplification protocol (LEAP) assays using L1 RNPs from HeLa cells indicated that overexpression of A3C reduces the L1 reverse transcription rate by approximately 50%, consistent with the observed inhibition of L1 retrotransposition by 40 to 60%. He proposed that A3C-mediated L1 restriction occurs via a cytidine deaminase-independent mechanism, based on an L1 mRNA-bridged interaction with L1 ORF1p, which inhibits the processivity of L1 reverse transcriptase (RT).

Retrogenes arise by L1-mediated retrotransposition of genomic DNA and are potentially imprinted (that is, expressed from only one of the two parental alleles, as a consequence of differential DNA methylation of the two alleles). **Mike Cowley** (laboratory of Rebecca Oakey, King’s College, London, UK) reported how imprinted retrogenes located within introns can control the choice of host-genes mRNA polyadenylation (poly(A)) sites. Transcription of the expressed retrogene correlates with host gene transcripts using poly(A) sites upstream of the retrogene insertion, whereas the silenced retrogene allele is associated with the use of downstream poly(A) sites. He proposed that intronic imprinted retrogenes might inflict alternative poly(A) sites on the host gene, possibly through a transcriptional interference mechanism, which may impact on the protein-coding capacity of the host gene. These intronic retrogenes also provide some of the first experimental examples of epigenetic marks influencing alternative polyadenylation.

Finally, **Constance Ciaudo** (laboratory of Olivier Voinnet, Ecole polytechnique fédérale de Zürich ETH-Z, Zürich, Switzerland) reported that L1 elements are regulated during mouse ES cell differentiation. L1 elements are strongly upregulated in RNA interference (RNAi) mutants at both mRNA and protein levels, and their copy number increases in Dicer mutants indicating an active retrotransposition. She demonstrated that Dicer is involved in regulation of L1 promoter activity. Using deep sequencing approaches, she reported the identification of small RNAs derived from young active L1 elements. These small RNAs are produced via RNA degradation pathways and are loaded into Argonaute complexes. She proposes that these small RNAs could be primal RNA involved in the epigenetic regulation of L1 elements.

Over the last decade, mechanisms of epigenetic regulation controlling TE activity have been extensively analyzed with numerous models species. Three talks focused on the silencing exerted on TEs in the germ line of *Drosophila melanogaster*, its differences with somatic lineages and its potential impact in the progeny.

**Yikang S. Rong** (National Cancer Institute, Bethesda, MD, USA) reported that *Drosophila* telomeric retroposons are an excellent system to study interactions between host and TEs. When analyzing their targeting to chromosome ends, he showed that these elements are under multiple modes of host regulation: in the germline, expression from the elements is suppressed by Piwi-interacting RNAs involved in the so-called piRNA pathway while in the soma, cell cycle-specific regulation limits their expression to a narrow window of the S-phase. Furthermore, the retrotransposon machinery forms large spherical structures encapsulating multiple chromosome ends suggesting an important role in telomere maintenance for the host.

**Kirsten-Andre Senti** (laboratory of Julius Brennecke, Institute of Molecular Biotechnology IMBA, Vienna, Austria) used tissue-specific RNAi to separately and specifically knockdown several piRNA pathway components in the *Drosophila* soma and germline. RNA sequencing (RNA seq) was then performed from ovary samples and from early embryos. This systematic analysis showed that the *Drosophila* piRNA pathway prevents access of TEs to the next generation, provides an annotation of the active elements repressed by the piRNA pathway, and will serve as a starting point to understand the complexity of the piRNA pathway at the transition between the female gonad and the next generation.

Although the piRNA pathway protects *Drosophila* against the deleterious effect of TE in the germline, **Emilie Brasset** (laboratory of Chantal Vaury, laboratoire de Génétique, Reproduction et Développement GReD, Clermont-Ferrand, France) reported the identification of a small window during cyst mitosis in the ovary, when Piwi expression is impaired. In these germinal cells, piRNA silencing is less efficient, offering a short period during which TEs can escape from host restraint and expand.

A paramutation is an interaction between two alleles of a locus, through which one allele induces a heritable modification of the other allele without modifying the DNA sequence. The group of **Stéphane Ronsseray** (University Pierre et Marie Curie, Paris, France), in collaboration with the group of Christophe Antoniewski (University Pierre et Marie Curie, Paris, France), reported a case of stable paramutation, which takes place in the *Drosophila* female germline. This paramutation was discovered using a homology-dependent silencing induced by *P*-element-derived transgenes and provides a genetic model for transgenerational repression of TEs.

In plants, the RNA-directed DNA methylation (RdDM) pathway triggers DNA methylation and gene silencing at repeated loci by 24-nt small interfering RNAs (siRNAs). **Thierry Lagrange** (Laboratoire Génome et Développement des Plantes, Perpignan, France) reported that ARGONAUTE4 binds 24-nt siRNAs and interacts with several RdDM components through evolutionarily conserved GW-rich motifs, called AGO hooks. Functional analysis of NERD, a novel AGO hook protein, demonstrated that *Arabidopsis* harbors a second siRNA-dependent DNA methylation pathway that targets a subset of loci, including non-annotated intergenic regions and TEs. The NERD pathway differs from RdDM by relying on 21-nt siRNAs and silencing-related factors so far implicated in post-transcriptional gene silencing (PTGS), including RNA-dependent RNA polymerase1/6 and ARGONAUTE2. Their results uncover a conserved chromatin-based RNA silencing pathway encompassing both PTGS and transcriptional gene silencing (TGS) components in plants that targets transposable elements.

## Session 3: Evolution of transposable elements

### Abdelkader Ainouche, Richard Cordaux, Cristina Vieira

TE evolution is strongly coupled to evolution of their host genomes. The session ‘Evolution of transposable elements’ included issues regarding features of host genomes related to TEs, the evolution of TE sequences, the way TEs have been horizontally transferred between organisms, and how TE evolution drives host evolution.

TEs very often behave as deleterious elements, and it is expected that selection will lead to their elimination from genomes. Although epigenetic mechanisms, such as DNA methylation, maintain a control over TE activity, it is less clear how epigenetic control of TEs may affect nearby genomic regions. **Brandon Gaut** (University of California, Irvine, CA, USA) showed that, in plants, methylation of genes flanking TEs is negatively correlated with their expression. TE silencing by methylation appears to be the cause of this phenomenon and, as such, methylation is a trade-off that benefits a plant by reducing TE activity but has the potential negative effect of perturbing gene expression. The implication of TE methylation on methylation of flanking genes was investigated by contrasting gene expression between orthologs that do or do not differ in the presence of a nearby (flanking) TE. The negative correlation between flanking methylation and gene expression appears to be a general feature of plant genomes, as demonstrated in grass (Poaceae) species. However, the strength of this correlation appears to vary between plant genomes, perhaps due to species differences in epigenetic response or strength of selection associated with that response. Interaction between TEs, methylation and gene expression likely plays a critical role in shaping the evolution of plant genome size and structure across angiosperms. Despite the fact that the mechanism that causes suppression of gene expression is unknown, it is possible that heterochromatic markers extend beyond the borders of some TEs to affect gene expression.

Another type of interaction between host genomes and mobile genetic elements was illustrated by **Harmit Malik** (Fred Hutchinson Cancer Research Center, Seattle, WA, USA), in the context of an evolutionary arms race between host and viral genomes. Interaction between poxvirus K3L and the host protein kinase R (PKR) was used as a model system for studying host proteins challenged by viral mimicry. Poxviral K3L mimics the conserved substrate of PKR (eIF2alpha). PKR has evolved under intense episodes of positive selection in primates, allowing it to evade antagonism by K3L. Thus, vaccinia K3L cannot defeat human PKR. In an experimental evolution system, vaccinia viruses rapidly acquired higher fitness via recurrent K3L gene amplifications to defeat PKR. These expansions also facilitated the gain of an adaptive amino acid substitution in K3L to defeat PKR. Poxviral ‘gene-accordions’ explains how poxviruses can rapidly adapt to defeat different host defenses despite low mutation rates.

**Aurélie Kapusta** (laboratory of Cédric Feschotte, University of Texas, Arlington, TX, USA) presented data on how bats may maintain relatively small genomes with high rates of DNA loss that counteracts TE invasions. While no activity has been reported in other mammals, vesper bats show recent waves of DNA transposon activity and therefore offer an unprecedented opportunity to study DNA transposon dynamics, regulation and genomic impact in one of the most extraordinarily species-rich group of mammals. Bats may keep their genomes slim thanks to a balance between moderate TE activity and high rate of DNA loss (via medium-size or large deletions). Intense genome contraction throughout bat evolution supports the idea that flight imposes a constraint on genome size.

Horizontal transfer of TEs between different species has often been reported and recent developments in sequencing technologies now allow us to test different hypotheses of gene transfer on an unprecedented scale. **Claudia Carareto** (Sao Paulo State University, Sao Paulo, Brazil) presented data on TEs from *Drosophila melanogaster* and *D. simulans* supporting the hypothesis of horizontal transfer between these two species. The full-length Bari and 412 copies in *D. melanogaster* appeared to be exclusively derived from *D. simulans* suggesting either that *D. melanogaster* did not inherit active copies from a common ancestor, or could have lost them early during its diversification. Bari and 412 appeared to have been reintroduced into *D. melanogaster* before the expansion of both species out of Africa (30 to 40,000 years ago), when *D. melanogaster* and *D. simulans* were in sympatry in mainland Africa. Species of the genus *Zaprionus* and of the melanogaster subgroup share the same age of origin and diversification in tropical Africa. The use of phylogenetic networks can resolve relationships among low diversity sequences, and can be used to infer the origin of multiple copies from a unique sequence, revealing relationships between ancestral and derived sequences.

The way TEs are horizontally transmitted between species is still a matter of discussion. Genome comparisons involving different interacting partners could give us indications on the way TEs are transferred. **Jean-Michel Drezen** (CNRS, University of Tours, Tours, France) discussed an illustration of this type of system. Functionally, bracovirus particles are gene transfer agents produced in the ovaries of parasitoid wasps. They are injected into the parasitized butterfly host and they ensure the production of virulence proteins by the host, altering its immune defenses. The DNA molecules packaged in the particles are produced from a proviral form integrated in the wasp genome, which constitutes a large target (≈1 megabase) for TE insertions. Remnants of retroelements and large DNA transposons (that is, a Maverick and several copies of a putative new element) were identified in the Cotesia congregata bracovirus (CcBV) genome. DNA circles packaged in the particles were recently shown to integrate into lepidopteran host DNA and analysis of a wasp genome revealed that reintegration of circles back into wasp DNA can also occur. Thus, bracovirus circles can navigate between genomes of different insect orders and are potential vehicles for genetic exchanges between genomes including TE transmission. Since several lepidopteran genomes have recently become available, it is now possible to evaluate the extent of bracovirus contribution to TE horizontal transfers between Hymenoptera and Lepidoptera.

The evolution of TEs can be very different depending on the host genomes. As reported by **Richard Cordaux** (CNRS, University of Poitiers, Poitiers, France), bacterial genomes usually experience high sequence turnover and short TE retention times, obscuring ancient TE evolutionary patterns. The genomes of six Wolbachia bacterial endosymbionts revealed the presence of numerous IS copies. IS account for >14% of *Wolbachia w*VulC genome, one of the highest IS genomic coverages reported in prokaryotes to date. Several processes may explain TE abundance in *Wolbachia*, including recent activity, along with recurrent invasions through horizontal transfers and gene conversion. Remarkably, 60 to 100% of IS within each *Wolbachia* genome are non-functional that is, have undergone or are undergoing decay. This can be explained by the particular lifestyle of *Wolbachia* endosymbionts, which exhibit reduced effective population sizes, relative to free-living bacteria, leading to relaxed efficiency of selection and enhanced genetic drift. The outcome for IS is fast fixation followed by slow removal via small deletions. This contrasts with free-living bacteria in which efficient negative selection leads to fast removal of IS. Non-functional IS constitute an unusual bacterial IS genomic fossil record providing direct empirical evidence for a long-term IS evolutionary dynamics following successive periods of intense transposition activity. Identification of an important IS genomic fossil record in *Wolbachia* demonstrates that IS are not always of recent origin, contrary to the conventional view of TE evolution in prokaryote genomes.

TE evolution and decay has also been documented in other genomes such as those of *Drosophila*. **Pierre Capy** (CNRS, Laboratoire Evolution, Génomes et Spéciation, Gif-sur-Yvette, France) presented an analysis of class II elements (DNA) indicating that most of the internal deletions that were identified in these TEs occurred between short regions (from 2 to 11 bp) exhibiting microhomologies. One hypothesis is that these internal deletions are due to errors in DNA double-strand break repair after excision of the TEs. It is tempting to propose that this system may be at the origin of miniature inverted-repeat transposable element **(**MITE**)** elements.

Analysis of other elements from *Drosophila*, the class I (RNA) HeT-A telomeric retrotransposons was reported by **Elena Casacuberta** (Institut de Biologia Evolutiva, Barcelona, Spain). Her results showed a 28-nucleotide sequence in the 3′ UTR region to be the most conserved sequence of the element within and among closely related species. This short sequence has been named HeT-A_pi1 since pairs of sense and antisense piRNA sequences have been detected in piRNA libraries of *D. melanogaster*. The high degree of conservation found in the piRNA target HeT-A_pi1 sequence suggests an important function of this sequence in the co-evolution of this TE and the *Drosophila* genome.

Small RNAs may play important functions in gene regulation in many other organisms. This was illustrated by **Eric Meyer** (CNRS, INSERM, Institut de Biologie de l’Ecole Normale Supérieure, Paris, France) for *Paramecium tetraurelia* mating type determination, one of the oldest known cases of ‘transgenerational epigenetic inheritance’. Mating type O is determined during development of the somatic macronucleus by the excision of the promoter of the mtA gene, which encodes a mating type E-specific transmembrane protein. Maternal inheritance of mating types is mediated by the scan RNA (scnRNA) pathway, a piRNA-like class of small RNAs required to eliminate TEs during macronuclear development. These RNAs target any sequence absent from the maternal macronucleus (through a process known as ‘genome scanning’) and assure their elimination. In the sibling species *Paramecium septaurelia*, mtA is also an E-specific protein and maternal inheritance of mating types is also due to a maternally inherited alternative rearrangement of the genome. However, the O type is determined by a deletion of part of mtB, encoding a transcription factor required for mtA expression, rather than by deletion of the mtA promoter. These examples illustrate the flexibility with which the scnRNA pathway is naturally used to maintain an essential phenotypic polymorphism in populations of genetically identical cells.

Long-term TE evolution can readily be reconstructed in mammals thanks to many degraded copies constituting genomic fossil records of past TE proliferations. **Astrid Engel** (Tulane University, New Orleans, LA, USA) reported the reconstruction of the consensus sequence of two ancient extinct L1 subfamilies from primate genomes (40 and 25 million years old) and demonstrated that they were both retrotranspositionally active and can support the retrotransposition of old and young Alu subfamilies at different levels of efficiency. The use of different cell lines showed that cellular factors significantly affect L1 retrotransposition efficiency of the older L1 element and that these factors have likely had a significant effect on L1 element evolution.

Also in primates, **Mojca Tajnik** (laboratory of Jernej Ule, University of Ljubljana, Ljubljana, Slovenia) reported that Alu exonization contributes to transcriptome diversity via alternative splicing. By using quantitative UV-crosslinking and immunoprecipitation (iCLIP) and RNA seq methods, the heterogenous nuclear ribonucleoprotein C1/C2 (hnRNP C) was identified as a silencer of antisense Alu element exonization. The mechanism of silencing was studied through competition of hnRNP C and the core splicing factor U2AF65 for binding on polypyrimidine tracts, using reporter minigene assays with introduced single-nucleotide substitutions and disease-related mutations in polypyrimidine tracts. This study revealed a new mechanism for maintaining transcriptome integrity via repression of the cryptic Alu exons.

## Session 4: Mechanisms of transposition

### Mireille Bétermier, Mick Chandler, Gaël Cristofari

The session on transposition mechanisms included talks, which addressed cellular control of transposon integration, transposase oligomerization and the control of transposition, mechanisms of reverse transcription and retrotransposition and transposase domestication and genome dynamics.

**Vincent Parissi** (CNRS, University of Bordeaux, Bordeaux, France) presented studies on the integration of human immunodeficiency virus (HIV) and prototype foamy virus (PFV) retroviruses. He described the effect of nucleosome positioning *in vitro* on concerted (double end) integration using purified components. HIV integration into stable nucleosomal regions was reduced, but this inhibition was partially alleviated by the ATP-dependent chromatin-remodeling complex SWI/SNF known to interact with the retroviral integrase IN. This is consistent with high throughput sequencing studies indicating that HIV integration is enriched in nucleosome-poor regions. On the other hand, integration of PFV was shown to be significantly less sensitive than HIV to the presence of stable nucleosomes. This pattern is consistent with the DNA bending (observed in structural studies) required for PFV integration but not for that of HIV.

Control of integration was also addressed by **Bernard Hallet** (University of Louvain, Louvain la Neuve, Belgium), for the bacterial Tn*4430* transposon, a member of the Tn*3* family whose transposase, like retroviral IN, belongs to the DDE superfamily. Tn*4430* transposes using a replicative cointegration mechanism and its insertion appears tightly coupled to target DNA replication. Slowing down the target replication fork results in preferential upstream insertions. Moreover, Tn*4430* transposase binds with high affinity to artificial DNA forks *in vitro* and uses these as a specific target for joining the transposon ends. The data suggest a ‘replication fork hijacking’ mechanism whereby Tn*4430* would recruit the cellular replication machinery by jumping into replication intermediates.

The theme was also taken up by **Bao Ton-Hoang** (CNRS, Laboratoire de Microbiologie et Génétique Moléculaires, Toulouse, France), who showed that the single strand DNA insertion sequence, IS*608*, which uses tyrosine rather than DDE chemistry for transposition, is excised from and inserts preferentially into the lagging strand template of both plasmid and chromosomal replication forks. Its transposase, TnpA, also localizes to blocked replication forks *in vivo* and preferentially binds branched DNA structures *in vitro*, such as forks, D-loops and Holliday junctions, suggesting a mechanism for assuring transposition activity at the fork.

Two presentations addressed the role of transposase oligomerization in regulating transposition. **Ronald Chalmers** (University of Nottingham, Nottingham, UK) described a model explaining overproduction inhibition (OPI), a phenomenon observed for several eukaryotic transposons where high levels of transposase result in a reduction in transposition activity. Using the human mariner TE, *Hsmar1*, he provided strong biochemical support for a model in which the key controlling element is a transposase dimer, which first binds a single transposon end via one of the component monomers before using the second to bind the other end to form an active transpososome. OPI is proposed to arise from occupation of each end of the transposon by a transposase dimer, preventing formation of the transpososome. The model was rigorously tested using computer simulations. It can be used to explain the strong tendency for TEs to undergo decay in eukaryotes.

**Fred Dyda** (National Institutes of Health, Bethesda, MD, USA) presented a set of crystallographic structures of the transposase of another eukaryotic TE, *Hermes* belonging to the *hAT* family. He identified an octomeric unit in which four dimers are intertwined via their C-terminal domains. This was found to have limited activity *in vitro*. However, the structure predicts that removal of an α-helix should destroy the interface holding the octomer together and result in the formation of transposase dimers. It was observed that such deletions render the transposase hyperactive *in vitro*. Although the exact details remain to be elucidated, it seems probable that the ‘closed’ octomeric form may represent a downregulated transposase species.

A third theme of this session was the mechanism(s) involved in reverse transcription and retrotransposition. **Thomas Eickbush** (University of Rochester, Rochester, NY, USA) presented the results of studies of R2 non-LTR retrotransposons. These elements integrate in a sequence-specific way into 28S RNA genes by target-primed reverse transcription (TPRT). R2 RNA, the transposition intermediate, is processed from a 28S/R2 cotranscript by a self-cleaving ribozyme located at the 5' R2 RNA end. Eickbush has now shown that this activity is present in all R2 elements from *Drosophila* to hydra. The position of cleavage varies from organism to organism and determines insertion site selectivity. When cleavage occurs within the 28S RNA moiety, subsequent insertion into a 28S DNA target is precise - presumably due to base pairing between the 28S gene and the remaining 28S RNA sequence attached to the R2 transcript. For those organisms in which cleavage occurs at the exact R2-28S RNA junction, insertion shows addition of non-templated nucleotides. Eickbush also localized by mutagenesis the RNA binding domain of the R2 protein in a region upstream of the reverse transcriptase domain conserved in other non-LTR retrotransposons and in telomerase.

In contrast to R2, mammalian LINEs, such as L1, do not integrate in a specific locus. Most L1 insertions occur in the highly frequent A/TTTT motif. However, individual sites are often degenerate and contain much longer stretches of AT-rich sequences. The L1 machinery is a ribonucleoprotein particle (RNP), which contains an additional RNA-binding protein, ORF1p, encoded by the L1 element. **Clement Monot** (laboratory of Gaël Cristofari, Inserm, CNRS, University of Nice-Sophia-Antipolis, Nice, France) has developed a direct reverse transcriptase assay with native L1 RNP for studying the initiation of reverse transcription. Using this system, he defined the preferential rules of L1 reverse transcription initiation. He showed that efficient priming can be achieved with as little as four matching nucleotides at the primer 3' end, but also that the L1 RNP can tolerate terminal mismatches if compensated by an increased number of upstream matching nucleotides. Based on these data, he proposed that the terminal bases of the primer act as a specific snap and the upstream ones as a weaker ‘velcro strap’ allowing efficient and flexible retrotransposition into imperfect AT-rich regions as observed in mammalian genomes.

**Alan Schulman** (Institute of Biotechnology, Helsinki, Finland) described *in vivo* studies with the *BARE* LTR-retrotransposons. These elements produce several RNA populations: one that is capped, polyadenylated, and translated but cannot be reverse transcribed; another that is not capped or polyadenylated, but packaged into virus-like particles (VLPs) and reverse transcribed; and a third which is capped, polyadenylated and spliced to produce high amounts of Gag, the capsid protein that forms the VLPs. The relative amount of the spliced and unspliced forms varies from tissue to tissue. These data highlight a unique situation among the retroelements where distinct RNA pools are committed to translation (with or without splicing) or reverse transcription depending on post-transcriptional processing.

The final theme of the session centered around transposase domestication and genome dynamics. **Bao Ton-Hoang** (Laboratoire de Microbiologie et Génétique Moléculaires, CNRS, Toulouse, France) presented evidence that an IS*608*-related tyrosine transposase has evolved to manage repeated extragenic palindromes (REP) sequences which are present in many bacteria in very high copy number and are involved in genome structure and gene expression. In *Escherichia coli*, only a single copy of the gene, *tnp*A_REP_, is present. It is located in an identical position in all strains. Phylogenetic evidence suggested that the gene arrived early in the radiation of *E.coli* and was later lost in some of the present clades. Purified TnpA_REP_ protein exhibited catalytic activity. It is capable of sequence-specific cleavage and strand transfer of REP sequences. This would provide one of the first examples of transposase domestication in a prokaryote.

Perhaps one of the most important examples of transposase domestication is that of the RAG proteins involved in generating V(D)J diversity. The RAG ancestor is thought to resemble the transposase of *Transib*. **Nancy Craig** (Johns Hopkins University School of Medicine, Baltimore, MD, USA) presented a functional analysis of the *Transib* transposase and demonstrated that, like V(D)J recombination, *Transib* transposition passes through an intermediate involving formation of a DNA hairpin on the flanking DNA.

The V(D)J system was addressed by **Martin Gellert** (National Institutes of Health, Bethesda, MD, USA), who presented data confirming that the ‘signal end’ complex within which the recombination reactions occur includes RAG1, RAG2, HMGB1 in a 2:2:1 stoichiometry. He also provided structural information from electron microscopy EM studies. This indicated a parallel anchor-shaped RAG1/2 complex with approximately 2-fold symmetry in which RAG2 is located at the head of anchor, RAG1 N-terminus at the ‘shank’ end, along with DNA chains beyond nonamers. Functional experiments showed that there is autoinhibition by interaction of RAG1 and RAG2 C-termini and that autoinhibition can be alleviated by binding a histone H3 tail peptide containing trimethylated lysine 4 (H3K4me3) and known to bind the PHD domain of RAG2 and to target it to recombinationally active loci. This is possibly the first known case where chromatin tethering activates an enzyme.

Ciliates have recently provided a novel illustration of the role played by domesticated transposases in developmentally programmed genome rearrangements. **Alexander Vogt** (laboratory of Kazufumi Mochizuki, Institute of Molecular Biotechnology (IMBA), Vienna, Austria), described Tpb2p, a domesticated *piggyBac*-like transposase essential for the excision of internal eliminated sequences (IESs), an obligatory genome rearrangement in the *Tetrahymena* life cycle. The enzyme introduces a 4-bp staggered cleavage at an IES boundary *in vitro*. Mutagenesis of the boundary sequence revealed a crucial role for positions 2 and 3 after the cut (*in vitro* and *in vivo*). In addition, IES-specific heterochromatin seems to control cleavage site accuracy through a possible interaction between Tpb2p and H3K9me3. Thus both boundary sequences and heterochromatin interactions are probably involved in specifying precise IES excision.

**Mireille Bétermier** (CNRS, Centre de Génétique Moléculaire, Gif-sur-Yvette, France) presented the analysis of a genome-wide set of IESs identified in *Paramecium* by high-throughput sequencing of DNA extracted from cells depleted in PiggyMac, the Tpb2p homolog responsible for IES excision in this ciliate. A vast majority of *Paramecium* IESs (93%) are shorter than 150 bp. Among the longest IESs, recognizable fragments of *Tc1*-like transposons were identified, indicating that PiggyMac excises DNA sequences unrelated to *piggyBac*. Again, this raises the question of how the domesticated transposase is targeted to its cleavage sites. The size distribution of *Paramecium* IESs exhibits a 10.2-bp periodicity that coincides with the helical phase of DNA. This may reflect DNA bending constraints on assembly of the IES excision complex. Evidence was presented for a requirement for Ku70/Ku80 before DNA cleavage, which would possibly favor the precise repair of IES excision sites by the NHEJ pathway.

### Next international meeting in 2014

The tradition of a large international meeting on Transposable Elements will continue in 2014, in the USA. The venue and dates will be announced soon.

## Abbreviations

Bp: base pair; ChIP-seq: chromatin immunoprecipitation followed by massive DNA sequencing; ERV: endogenous retroviruses; ES cell: embryonic stem cell; iCLIP: UV-crosslinking and immunoprecipitation; IES: internal eliminated sequence; IS: insertion sequence; LEAP: L1 element amplification protocol; L1: LINE-1; LTR: long terminal repeat; ORF: open reading frame; MITE: miniature inverted-repeat transposable element; NAHR: non-allelic homologous recombination; NER: nucleotide excision repair; NGS: next-generation sequencing; OPI: overproduction inhibition; piRNA: Piwi-interacting RNAs; PTGS: post-transcriptional gene silencing; RdDM: RNA-directed DNA methylation; REP: repeated extragenic palindromes; RIP: retrotransposon insertion polymorphism; RNP: ribonucleoprotein; RNAi: RNA interference; RNA seq: RNA sequencing; RT: reverse transcriptase; scnRNA: scan RNA; SHAPE: selective 2′-hydroxyl acylation analyzed by primer extension; siRNA: small interfering RNA; TAP: tandem affinity purification; TPRT: target-primed reverse transcription; TE: transposable element TGS, transcriptional gene silencing; TSS: transcription start site; UTR: untranslated region; VLP: virus-like particle.

## Competing interests

The authors declare that they have no competing interests.

## Authors’ contributions

J-MD wrote Introduction and keynote lecture. OP, HQ and ClV wrote Session 1: Impact on genomes, PL and ChV wrote Session 2: Control of transposition, AA, RC and CrV wrote Session 3: Evolution of transposable elements, MB, MC and GC wrote Session 4: Mechanisms of transposition. All authors read and approved the final manuscript.

## Funding

Work in the laboratory of M. Bétermier is supported by intramural funding from Centre National de la Recherche Scientifique (CNRS) and by the Agence National de la Recherche (ANR) (grants ANR BLAN08-3-310945 ‘ParaDice’ and ANR 2010-BLAN-1603 ‘GENOMAC’). Work in the laboratory of M. Chandler is supported by intramural Centre National de la Recherche Scientifique (CNRS) funding and by ANR grant MOBIGEN. Work in the laboratory of G. Cristofari is supported by grants from the Institut National de la Santé et de la Recherche Médicale (INSERM) and the Institut National du Cancer (INCa, Avenir 2008 Grant), and the European Research Council (ERC number 243312, Retrogenomics). Work in the laboratory of P. Lesage is supported by intramural CNRS and INSERM funding. Work in the laboratory of C. Vieira is supported by the ANR Genemobile and the Institut Universitaire de France. Work in the laboratory of C. Vaury is supported by grants from INSERM, CNRS, the Université d’Auvergne and Région Auvergne.

